# 

**Published:** 1871-08

**Authors:** 


### INDEX OF SUBJECTS.

ORIGINAL COMMUNICATIONS.

Hydrate of Chloral....................................................398

Enemata....................................................405

Dysentery--its Treatment....................................................411

EXTRACTS AND GLEANINGS.

Bromide of Quinine, Morphine and Strychnine, 428; Condurango--Nitric Acid in Bright's Disease—Chlor-Alum for unbroken Chilblains, 429; Solution of Ammonia in Delirium Tremons—To prevent pitting in Smill Pox, 430; Belladonna in the treatment of Spermatorrhœa—Treatment of Chilblains—Com. Solution of Iodine in Chronic Diarrhœa—Belladona in Typhoid fever, 431; Hydrate of Chloral in Intermittent fever—Therapeutic Actions and Uses of Turpentine—Soda-mint, 432; Carbolic Cetrate—The After-taste of Quinine—Artificial Food for Infants, 433; Chloral in Cancer—Injections of Chloral in Syphilis—Treatment of Inverted Toe-nail, 434; Dressing of Wounds with Tinfoil—Management of Scabies—Castor Oil made Palatable, 435; Rigid os Uteri—Glyerine inhalations in Croup—Chloral Hydrate in Enuresis--Hydrate of Chloral in Whooping Cough, 436; Cod Liver Oil made Palatable--Treatment of Enlarged Tonsils—Iodide of Ammonia preferred to Iodide of Potassium, 437; Hypodermic Medication—A New Test for Hysteria, 438; Treatment of Intermittent Fever, 439; Salicine in Typhoid Fever—Veratum Viride in Peueperal Convulsions, 440; Treatment of Varicose Viens—Acute Inflammation of the Prostrate Glands, 441; Painful Menstrnation, 442.

CORRESPONDENCE—HYGENIC AND MISCELLANEOUS.

Sleeplessness in Infants....................................................443

EDITORIALS, REVIEWS, ETC.

To Our Subscribers—Our Associated Editors....................................................446
Extract of Letter from a Correspondent....................................................447
Books and Pamphlets....................................................450
College Announcements....................................................453
Editorial....................................................455

